# Supplementary material for: Predicting visual function by interpreting a neuronal wiring diagram
Source: Nature. 2024 Oct 2;634(8032):113–23. doi: 10.1038/s41586-024-07953-5 (PMC11446822; doi:10.1038/s41586-024-07953-5)
Supplement: Supplementary file 6 — CRF and ERF predictions for individual TmY4 and TmY9 cells. Analogous to Supplementary Data 3, but for TmY target types. Shown are the top four monosynaptic pathways, the strongest pathway passing through each of the top ten intermediary types (ranking from Extended Data Fig. 7), and the trisynaptic pathway Tm1–TmY–Dm3–TmY (see the section entitled Prediction of spatial normalization). [file 41586_2024_7953_MOESM6_ESM.zip › DataS4/TmY9q/720575940606178994.pdf]

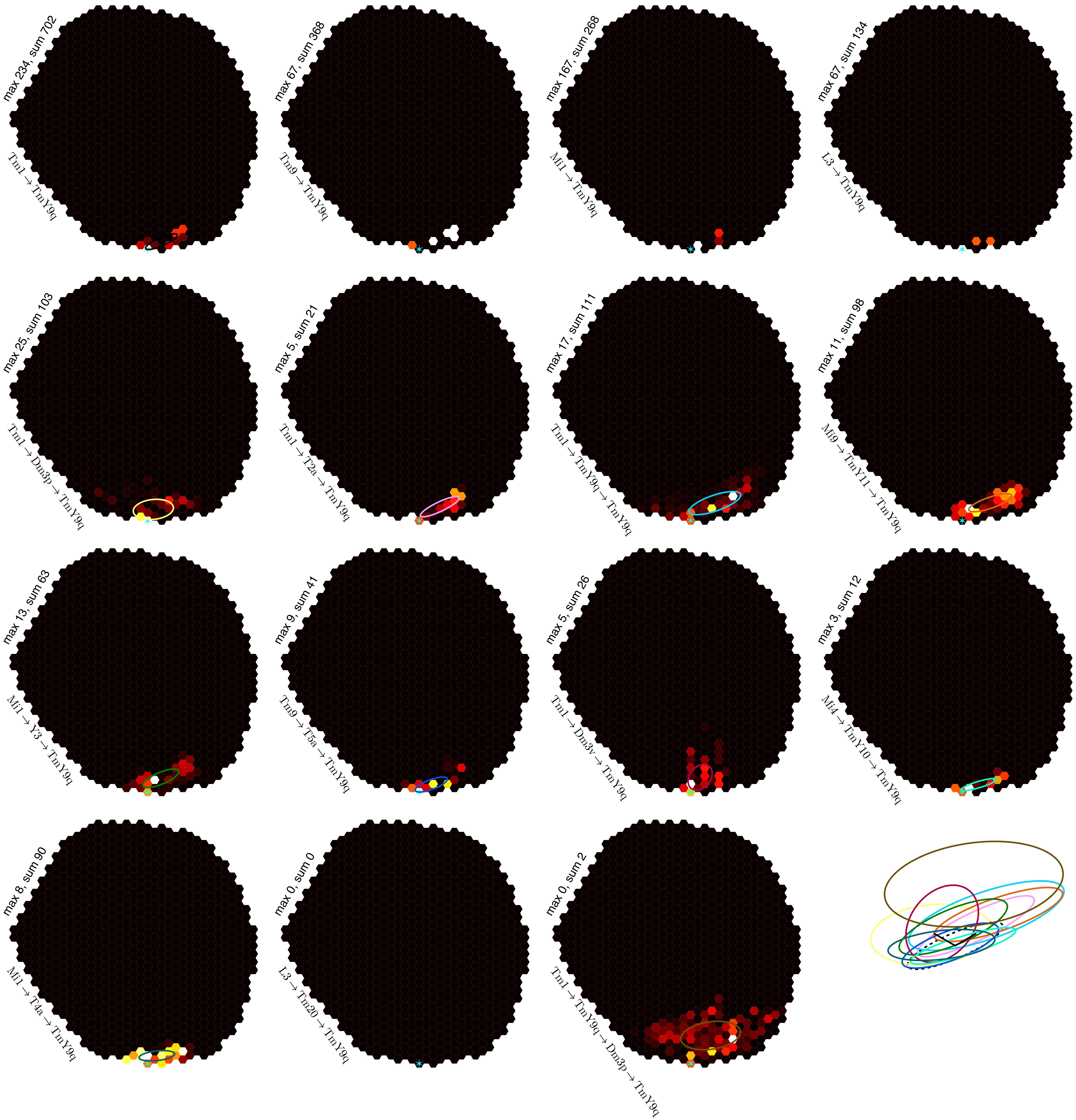
max 234, sum 702
Tm1 → TmY9q
max 67, sum 368
Tm9 → TmY9q
max 167, sum 268
Mi1 → TmY9q
max 67, sum 134
L3 → TmY9q
max 25, sum 103
Tm1 → Dm3p → TmY9q
max 5, sum 21
Tm1 → T2a → TmY9q
max 17, sum 111
Tm1 → TmY9q → TmY9q
max 11, sum 98
Mi9 → TmY11 → TmY9q
max 13, sum 63
Mi1 → Y3 → TmY9q
max 9, sum 41
Tm9 → T5a → TmY9q
max 5, sum 26
Tm1 → Dm3v → TmY9q
max 3, sum 12
Mi4 → TmY10 → TmY9q
max 8, sum 90
Mi1 → T4a → TmY9q
max 0, sum 0
L3 → Tm20 → TmY9q
max 0, sum 2
Tm1 → TmY9q → Dm3p → TmY9q
